# 463. Comparative Effectiveness of Dihydroartemisinin-Piperaquine and Sulfadoxine-Pyrimethamine in Preventing Adverse Pregnancy Outcomes: A Meta-Analysis and Systematic Review

**DOI:** 10.1093/ofid/ofaf695.155

**Published:** 2026-01-11

**Authors:** Jose Luis Boene, Sophia Costa, Oscar Hernández Rios, Thiago Netto, Taniela M Bes, Clarisse Bressan

**Affiliations:** Eduardo Mondlane University, Maputo, Maputo, Mozambique; Jose Lucas Municipal Hospital, Belo Horizonte, Minas Gerais, Brazil; Ricardo Palma University, Lima, Lima, Peru; Evandro Chagas National Institute of Infectious Diseases (Fiocruz), Rio de Janeiro, Rio de Janeiro, Brazil; MetroWest Medical Center, Framighan, MA; Evandro Chagas National Institute of Infectious Diseases (Fiocruz), Rio de Janeiro, Rio de Janeiro, Brazil

## Abstract

**Background:**

Malaria in pregnancy remains a significant public health challenge, particularly in sub-Saharan Africa (SSA), where it contributes to maternal anemia, low birthweight (LBW), preterm birth (PTB), and neonatal mortality. With increasing resistance to sulfadoxine-pyrimethamine (SP), the World Health Organization (WHO)-recommended intermittent preventive treatment during pregnancy (IPTp) strategy faces declining efficacy. Dihydroartemisinin-piperaquine (DHA-PQ) has emerged as a promising alternative, but its impact on broader perinatal outcomes remains unclear.

**Figure:**
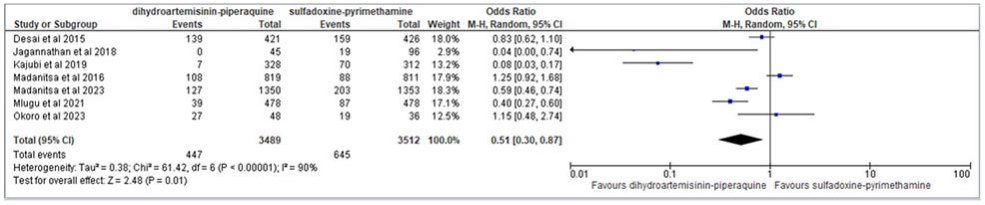
Placental malária (microscopy, RDT, LAMP, PCR)

**Figure:**
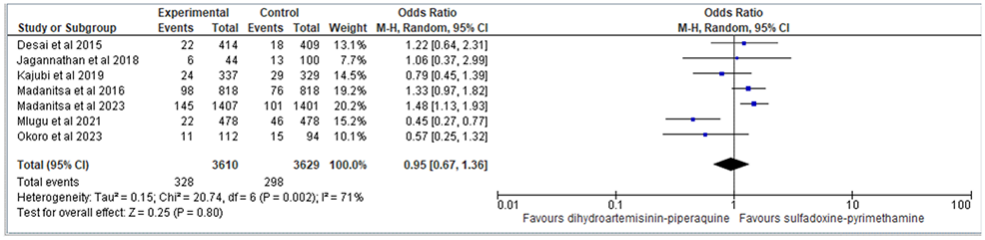
Low Birth Weight

**Methods:**

A systematic search of PubMed, EMBASE, Cochrane Library, and LILACS was conducted to identify randomized controlled trials (RCTs) comparing IPTp-DHA-PQ to IPTp-SP. Outcomes included placental malaria, Low birth weight (< 2500 g), fetal loss (still birth and spontaneous abortion), Pre Term Birth (< 37 weeks), neonatal death (0–28 days), and fetal anemia (Hb < 11 g/dL) . Data were pooled using a random-effects meta-analysis, with odds ratios (OR) and 95% confidence intervals (CI) reported. Risk of bias was assessed using Cochrane methods, and heterogeneity was evaluated using the I² statistic.

**Figure:**
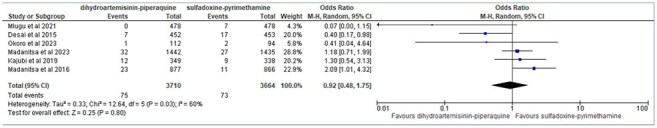
Fetal loss (still birth and spontaneous abortation)

**Figure:**
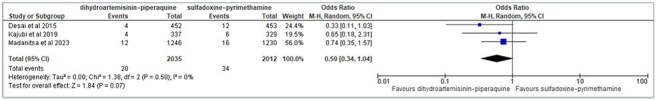
Neonatal Deaths

**Results:**

Seven RCTs involving 8,080 participants were included. DHA-PQ significantly reduced the incidence of placental malaria compared to SP (OR: 0.51, 95% CI: 0.30–0.87), demonstrating superior efficacy against malaria-specific endpoints. However, no significant differences were observed between DHA-PQ and SP for LBW, PTB, fetal loss, or neonatal deaths. Both regimens were well-tolerated, with minimal adverse effects reported.

**Conclusion:**

DHA-PQ effectively prevents placental malaria, particularly in regions with high SP resistance, while showing comparable outcomes to SP for broader pregnancy complications. These findings support its use as an alternative to SP and highlight the need for integration into holistic antenatal care strategies to improve pregnancy outcomes in malaria-endemic regions, informing potential updates to WHO guidelines.

**Disclosures:**

All Authors: No reported disclosures

